# A Lifestyle Modification Program for Secondary Prevention of Atrial Fibrillation: A Pilot Study

**DOI:** 10.21203/rs.3.rs-3369346/v1

**Published:** 2023-09-26

**Authors:** Jeffrey M. Ashburner, Taylor D. Carmichael, Romit Bhattacharya, Aneesh C. Bapat, Pradeep Natarajan, Steven J. Atlas, Daniel E. Singer, Anne N. Thorndike

**Keywords:** atrial fibrillation, lifestyle modification, secondary prevention

## Abstract

**Background:**

Lifestyle modification programs, such as cardiac rehabilitation, may reduce atrial fibrillation (AF) burden and improve quality of life (QOL), but remain unproven. The objective of this pilot study was to assess feasibility, acceptability, and preliminary effectiveness of an exercise and nutrition-based cardiac rehabilitation-like program for AF patients.

**Methods:**

We enrolled overweight adults aged ≥ 30 years with symptomatic AF in a 12-week cardiac lifestyle group program, including 6 virtual and 6 in-person visits. All visits included discussion and education about nutrition, exercise, and behavior modification. In-person visits included supervised aerobic exercise and strength training. Outcomes at baseline and 12 weeks included feasibility of participation, acceptability, change in weight and BMI, and changes in survey-based AF burden, symptoms, and QOL.

**Results:**

From 84 invitees, 11 (13.1%) were enrolled (mean age 64; baseline BMI 38 kg/m^2^); 9 (82%) completed the program. Patients attended an average of 9.7 (81%) visits (Range: 6–11). Mean weight loss was 9.1 pounds (Range: 0–16); mean BMI decrease was 1.4 kg/m^2^ (Range: 0–2.6). Patients found the program helpful overall: all reported making diet and exercise changes during the program. Compared to baseline, patients reported decreased AF burden (12.9 vs. 11.7, p = 0.03) and symptom (10.1 vs. 5.6, p = 0.003) scores at the conclusion of the program. Patients also reported increased QOL overall (68.9 vs. 86.4, p = 0.001)

**Conclusions:**

Participation in a cardiac rehab-like program was feasible and acceptable for overweight patients with symptomatic AF. Results suggest preliminary effectiveness of the program for reducing AF burden and symptoms and increasing QOL.

## Introduction

Atrial fibrillation (AF) is the most common cardiac arrhythmia, is associated with a 5-fold increased risk of stroke, a 1.5–2 fold increase in all-cause mortality, and is further associated with significant morbidity and risk of hospitalization.^1–4^ Lifestyle modification may represent an opportunity to improve management of this chronic condition.

Lifestyle modification programs, such as cardiac rehabilitation, may reduce AF symptoms and progression, as well as improve quality of life, but remain unproven.^5^ The American Heart Association has identified lifestyle modification in AF as an important therapeutic avenue that has been “underrecognized, underused and understudied” and noted the need for improved implementation strategies to deliver these therapies.^5^ Despite promising evidence, exercise-based cardiac rehabilitation is not part of routine care for patients with AF.^5^ Existing studies of lifestyle modification and secondar prevention of AF have been small, largely short-term, and may not be sustainable for subjects after the research study ends.

In this pilot study, we examined the feasibility, acceptability, and preliminary effectiveness of an exercise and nutrition-based cardiac lifestyle behavioral program for patients with symptomatic AF. We plan to conduct a future pragmatic randomized trial of patients with symptomatic, paroxysmal AF to a cardiac lifestyle program compared to usual care. In this trial, we will examine the effectiveness of the intervention on decreasing AF burden and symptoms, as well as how well the benefits of the intervention persist over a 12-month period.

## Methods

### Design and setting

Eligible patients were overweight or obese (body mass index [BMI] ≥ 28 kg/m^2^) adults aged ≥ 30 years with symptomatic paroxysmal AF seen by an electrophysiologist at Massachusetts General Hospital. Patients were excluded if asymptomatic, scheduled for catheter ablation, or if their physician did not think they could participate in an exercise program. Potentially eligible patients were identified from an electronic health record query. Research staff conducted an initial chart review to remove those not eligible, and final eligibility was confirmed by the treating physician.

For patients not excluded by their physician, we sent an introductory letter and study information sheet with an option to opt out. A week later, a research assistant contacted patients by phone to assess interest in study participation. Interested patients completed informed consent.

### Intervention

Following consent, patients were enrolled in a 12-week cardiac lifestyle group program, modeled from cardiac rehabilitation^5^, which included 6 virtual and 6 in-person visits. This clinical program was available as part of routine care to patients with metabolic syndrome but was not available solely because of an AF diagnosis. Consented patients were scheduled for an initial in-person evaluation with a team physician, that included: detailed history and physical exam, review of cardiac risk factors and cardiac exam, nutritional assessment, comprehensive weight and diet history, exercise history, lipid profile, body composition assessment, and determination of percent body fat and BMI.

Visits during the 12-week program included 1-hour of group discussion and didactic education about nutrition, exercise, and behavior modification led by a physician and a dietitian. In-person visits also included 1-hour of supervised aerobic exercise and strength training.

### Outcomes

All outcomes were assessed at 12 weeks. Primary outcomes were feasibility and acceptability. Feasibility outcomes included the proportion of study visits completed (mean, median, minimum, maximum), the proportion of patients who completed the program, and the proportion of eligible patients who expressed interest in participating. Acceptability was assessed by survey following completion of the program and included questions about satisfaction with the program overall and with specific program components.

Secondary preliminary effectiveness outcomes included changes from baseline in weight, BMI, survey-based AF burden and symptoms^6^, and quality of life (QOL)^7^. AF burden and symptoms was assessed at baseline and follow-up by survey using the Atrial Fibrillation Severity Scale (AFSS),^6^ and quality of life was assessed with the AF Effect on Quality-Of-Life Questionnaire (AFEQT).^7^ Changes in outcomes from baseline were evaluated with paired sample t-tests. The sample size goal was 10–12 patients. The research protocol was approved by the Mass General Brigham Institutional Review Board. Participants provided written informed consent to participate.

## Results

**Figure 1:**
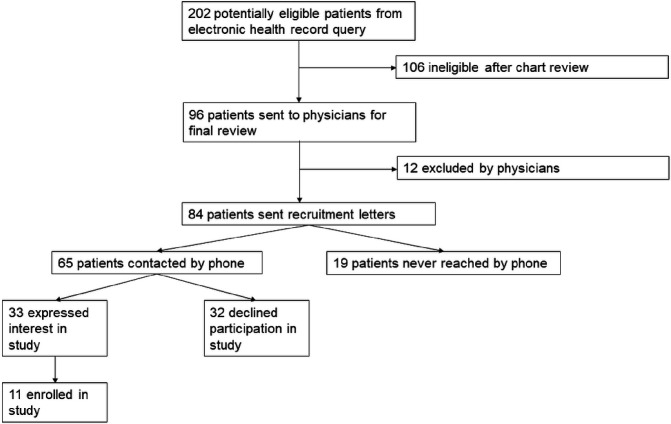
Flow diagram of patient identification, eligibility confirmation, recruitment, and enrollment

Among 96 potentially eligible patients, 12 (13%) were excluded by their physician. Among 84 patients sent recruitment letters, 33 (39%) expressed interest in the program, 32 (38%) declined participation, 19 (12%) were never reached by phone, and 11 (13%) enrolled (Fig. 1). Recruitment was stopped at 11 due to program capacity. The mean age of enrollees was 64.2 years, 5 (46%) were female, 11 (100%) were White, the mean BMI was 38.2 kg/m^2^ (Range: 29.9–48.9 kg/m^2^), and 8 (72%) were anticoagulated at baseline. Of 11 enrollees, 9 (82%) completed the program and all study surveys. Among those who completed the program, the mean number of visits attended was 9.7 of 12 (81%) (Median: 10, Range 6–11).

Patients found the program helpful overall, and all reported making diet and exercise changes (Table 1). Patients indicated that reviewing program notes/handouts (78%), periodic check-ins with program staff (67%), and logging diet and exercise daily (67%) would help maintain lifestyle changes.

Mean weight loss in those completing the program was 9.1 pounds (Range: 0–16 pounds, p = 0.002); mean BMI decreased 1.4 kg/m^2^ (Range: 0–2.6 kg/m^2^, p = 0.002). Compared to baseline, patients reported decreased AF burden (12.9 vs. 11.7, p = 0.03) and symptom (10.1 vs. 5.6, p = 0.003) scores at the conclusion of the program. Patients also reported increased QOL overall (68.9 vs. 86.4, p = 0.001) and for all QOL subscales: symptoms (85.2 vs. 92.6, p = 0.05), daily activities (60.2 vs. 84.2, p < 0.001), treatment concerns (67.6 vs. 85.2, p = 0.004) (Table 2).

## Discussion

Prior to conducting a large-scale pragmatic trial of an exercise and nutrition-based cardiac lifestyle modification program for secondary prevention of AF, this pilot study demonstrated that participation in a lifestyle modification program was feasible and acceptable for overweight and obese patients with symptomatic AF. Importantly, results suggest preliminary effectiveness of the program for reducing AF burden and symptoms and increasing QOL.

### Comparisons to existing research

There is growing evidence that lifestyle modifications have a significant impact on management of AF.^5^ Randomized studies examining the impact of lifestyle modification and cardiac rehabilitation on secondary prevention of AF have been limited in size and duration, with few evaluating sustainability.^8^ The ACTIVE-AF randomized trial demonstrated that a tailored aerobic-based exercise program reduced AF symptom severity and improved maintenance of sinus rhythm.^9^ These benefits were achieved in the absence of weight loss. Other studies have reported decreased AF burden and symptoms with aggressive risk factor reduction, including weight loss.^10,11^ Interventions evaluating the benefits of interventions that include both exercise and nutrition components warrant further investigation. Our pilot trial demonstrated feasibility and preliminary effectiveness of a hybrid, health clinic and virtual home-based, intervention to promote lifestyle modification related to both exercise and nutrition for secondary prevention of AF. Such a model may increase participation and be more sustainable than an entirely facility-based approach.^12^ Future research should evaluate the efficacy of a hybrid-based exercise and nutrition-based program for AF related (symptoms, recurrence, progression) and clinical (hospitalizations, mortality) endpoints in a multi-site, adequately powered clinical trial.

### Strengths and limitations

There was significant interest in study participation among eligible patients. More than half the patients reached by phone expressed interest in participating in the study and we quickly met our sample size goal. Among our 11 enrolled subjects, there was good retention with 9 completing the full 12-week program.

Our sample size of 11 enrolled subjects is small. Available funding for this pilot study was not significant enough to expand capacity of this clinical program. While our sample did have adequate representation of women (n = 5 of 11), we did not have an ethnically or racially diverse sample and did not have information about educational attainment or income. Additionally, we were unable to evaluate long-term sustainability of changes in diet and/or exercise beyond the 12-week program, or maintaining improvements in AF burden/symptoms and QOL. However, evaluating sustainability is a goal of the larger trial.

## Conclusions

This study demonstrates that enrolling overweight and obese patients with symptomatic AF in an exercise and nutrition-based cardiac lifestyle modification program for secondary prevention of AF is both acceptable to patients and feasible. An adequately powered pragmatic randomized trial comparing such a program to usual care appears possible.

## Figures and Tables

**Table 1:** Program acceptability survey completed after the 12-week program

Survey Components*	Strongly Agree	Agree	Neutral	Disagree or Strongly Disagree
I found the Cardiac Lifestyle Program helpful overall	8 (89%)	1 (11 %)	0 (0%)	0 (0%)
I found the in-person group sessions helpful	7 (78%)	1 (11 %)	1 (11 %)	0 (0%)
I found the virtual group sessions helpful	6 (67%)	2 (22%)	1 (11 %)	0 (0%)
I felt comfortable completing supervised exercises	6 (67%)	3 (33%)	0 (0%)	0 (0%)
I felt comfortable completing exercises on my own at home or at a gym	5 (56%)	4 (44%)	0 (0%)	0 (0%)
I am confident I will be able to continue changes to my diet after the program	5 (56%)	3 (33%)	1 (11 %)	0 (0%)
I am confident I will be able to continue changes to my exercise habits after the program	3 (33%)	4 (44%)	1 (11 %)	1 (11 %)
Continuing to follow the exercise and diet choices I developed during this program will help with my AF symptoms	6 (67%)	2 (22%)	1 (11 %)	0 (0%)
Continuing to follow the exercise and diet choices I developed during this program will improve my overall health	7 (78%)	1 (11 %)	1 (11 %)	0 (0%)

* All measures are from 9 of 11 patients who completed the program

**Table 2:** Patient reported AF burden, symptoms, and quality of life at baseline and follow-up

	Baseline (n = 9)	Follow-Up (n = 9)	Mean Difference	p-value
**Atrial Fibrillation Severity Scale (AFSS)**				
Burden (Scale Range: 3–30)	12.9	11.7	−1.2	0.03
Symptoms (Scale Range: 0–35)	10.1	5.6	−4.6	0.003
**Atrial Fibrillation Effect on Quality of Life (AFEQT)**				
(Scale Range: 0–100)*				
Overall	68.9	86.4	17.5	0.001
Symptoms	85.2	92.6	7.4	0.052
Daily Activities	60.2	84.2	24.1	<0.001
Treatment	67.6	85.2	17.6	0.004

* Higher scores indicate increased QOL

## Data Availability

The datasets generated during and/or analyzed during the current study are available from the corresponding author (J.M.A.) on reasonable request.

## Abbreviations

AF: atrial fibrillation
BMI: body mass index
QOL: quality of life
AFSS: Atrial Fibrillation Severity Scale
AFEQT: AF Effect on Quality-Of-Life Questionnaire
